# Molecular and cellular basis for immunomodulation with monoclonal antibodies

**DOI:** 10.1186/1479-5876-13-S1-K17

**Published:** 2015-01-15

**Authors:** Ignacio Melero

**Affiliations:** 1 CIMA, CUN and Medical School, University of Navarra, Pamplona, Spain

## 

Immunotherapy with immunostimulatroy monoclonal antibodies is based on the ability of antibodies to act as antagonists of inhibitory receptors or to act as agonists of costimulatory receptors. Interference of ligand to receptor binding underlies the first function while crosslinking of receptors to enforce signaling involves the second mode of action. Signalling receptors on lymphocytes and antigen presenting cells are embedded in the plasma membrane and are functionally linked to adaptors whose function is conducive to intracelluar signaling events.

In the case of antibodies blocking CTLA-4 functions multiple mechanism of action take place: (i) efficient competition for costimulatory interactions of CD28 with the very same ligands (CD80 and CD86), (ii) functional expression on the surface of regulatory T cells that can be depleted by ADCC, (iii) prevention of the denudation by cooption of CD80 molecules from the surface of antigen presenting cells, a(iv) interference with putative negative signalling mediated by phosphatases. The restricted pattern of expression of CTLA-4 on Tregs and activated T cells is critical to understand its function and exploitation in immunotherapy.

PD-1 upon interaction with PD-L1 or PD-L2 drives to the immune synapse the tyrosine phosphatases SHP-1 and SHP-2. These phosphatases, that are recruited to the cytoplasmic tail of PD-1 through its phosphorylated ITIM motive, dephosphorylate critical tyrosine residues in signaling adaptors activated by the concerted action of the TCR and CD28. Disruption of signalling in the immune synapse is prevented by blocking antibodies that avoid the guidance of PD-1 moieties to the synapse guided by PD-L1. Avoiding disruption of productive immune synapses is probably the main mechanism of action of anti-PD-1 and anti-PD-L1 blocking mAb.

The costimulatory members of the TNFR family transduce costimulatory signals by recruitment of the adptors TRAF-1, -2, -5. Signal transduction enhances NF-κB and MAPK signaling routes. In this pathway K-63 polyubiquitination reactions are critical for early signaling and are regulated by specific deubiquitinases. Receptors signal depending on crosslinking by the agonist antibody. The pattern of expression of these receptors on the different subset resting or primed/activated T cells determines function.

Overall precise understanding of the pattern of distributions of the receptors on immune cells and the signaling events that these receptors prevent or elicit will be ultimately important not only to understand the function but also for both therapeutic manipulation and biomarker finding.

